# How neurogenesis finds its place in a hardwired sensory system

**DOI:** 10.3389/fnins.2014.00102

**Published:** 2014-05-09

**Authors:** Livio Oboti, Paolo Peretto

**Affiliations:** ^1^Children's National Health System, Center for Neuroscience ResearchWashington, DC, USA; ^2^Department of Life Sciences and Systems Biology, Neuroscience Institute Cavalieri Ottolenghi, University of TorinoOrbassano, Italy

**Keywords:** accessory olfactory bulb, AOB, vomeronasal, VNO, neurogenesis, pheromones, plasticity, innate

## Abstract

So far most studies on adult neurogenesis aimed to unravel mechanisms and molecules regulating the integration of newly generated neurons in the mature brain parenchyma. The exceedingly abundant amount of results that followed, rather than being beneficial in the perspective of brain repair, provided a clear evidence that adult neurogenesis constitutes a necessary feature to the correct functioning of the hosting brain regions. In particular, the rodent olfactory system represents a privileged model to study how neuronal plasticity and neurogenesis interact with sensory functions. Until recently, the vomeronasal system (VNS) has been commonly described as being specialized in the detection of innate chemosignals. Accordingly, its circuitry has been considered necessarily stable, if not hard-wired, in order to allow stereotyped behavioral responses. However, both first and second order projections of the rodent VNS continuously change their synaptic connectivity due to ongoing postnatal and adult neurogenesis. How the functional integrity of a neuronal circuit is maintained while newborn neurons are continuously added—or lost—is a fundamental question for both basic and applied neuroscience. The VNS is proposed as an alternative model to answer such question. Hereby the underlying motivations will be reviewed.

## Introduction

The idea of a mature brain as an organ with limited growth, cell renewal and rewiring has considerably changed since pioneer studies on adult neurogenesis (Altman and Das, 1966; Altman, 1969; Graziadei and Monti-Graziadei, 1979; Lledo and Gheusi, 2006; Kempermann, 2012). In the adult mammalian brain, neural progenitors are present in the subventricular zone (SVZ) of the lateral ventricles and the hippocampal subgranular zone (SGZ) where they give rise respectively to Dlx2/5/6-derived GABAergic olfactory bulb interneurons and glutamatergic granule cells of the dentate gyrus (DG) of the hippocampus (Doetsch et al., 1999; Seri et al., 2001; Kriegstein and Alvarez-Buylla, 2009). Admittedly, neurogenesis in other adult brain regions is generally believed to be very limited under physiological conditions (Nishiyama et al., 1996; Horner et al., 2000; Dawson et al., 2003; Luzzati et al., 2006, 2011; Bonfanti and Peretto, 2011), although it could be induced after injury (Ramaswamy et al., 2005; Gould, 2007; Yu et al., 2008; Kernie and Parent, 2010; Saha et al., 2013) or as a consequence of tissue inflammation and degeneration (Buffo et al., 2008; Ohira et al., 2010; Luzzati et al., 2011; Belarbi and Rosi, 2013). Nowadays several approaches have been developed to maintain and manipulate pluripotent stem cells *in-vitro* in the perspective of brain repair (Takahashi and Yamanaka, 2006; Yamanaka and Blau, 2010). Particularly the rodent olfactory bulb (OB) has been widely studied to clarify the logic of neuronal stem-cell biology in the SVZ opening new venues to brain-repair strategies, cell transplants techniques in disease models and other translational approaches (Gage and Temple, 2013).

However, the development of clinical translations cannot stand aside the basic research, focused in this case on the physiologic function of the neurogenic regions *in-vivo* (see for critical reviews on this point Lau et al., 2008; Lindvall and Kokaia, 2010).

In addition, studying OB neurogenesis may yield new insights in the biology of olfaction, being olfactory sensory activity and behaviors easy readouts of any experimental manipulation in rodents.

Understanding how the environment affects newborn neurons integration into mature networks, and consequently normal brain function, are certainly meaningful aims to define the boundaries between physiology and pathology in translational neuroscience. The restoration of brain connectivity after trauma or the comprehension of the etiology of major brain disorders may certainly move forward and undoubtedly more clinically oriented approaches would benefit from the unbiased attempts of basic research to address these issues (Fang and Casadevall, 2010; Enserink, 2013). In the present manuscript a special attention will be given to neurogenesis in a particular olfactory subsystem—namely the accessory/vomeronasal system (VNS)—due to the fact that, despite its behavioral relevance in rodent sociality, it received so far a minor deal of attention. The main point hereby stressed concerns the unclear relationship between form—plastic and changing—and function—presumably stable, innate—in the VNS. Due to its distinct peculiarities, compared to the rest of the olfactory system, the VNS offers an unparalleled opportunity to analyze how newborn neurons constantly integrate into mature circuits without interfering with the physiological behavioral and endocrine development. Recent findings on neurogenesis in the vomeronasal organ (VNO) and accessory olfactory bulb (AOB) will be listed and discussed with a particular emphasis on the AOB, since it represents the first central brain region of this olfactory pathway.

## The vomeronasal system as a model to study adult neurogenesis

**Figure 1:**
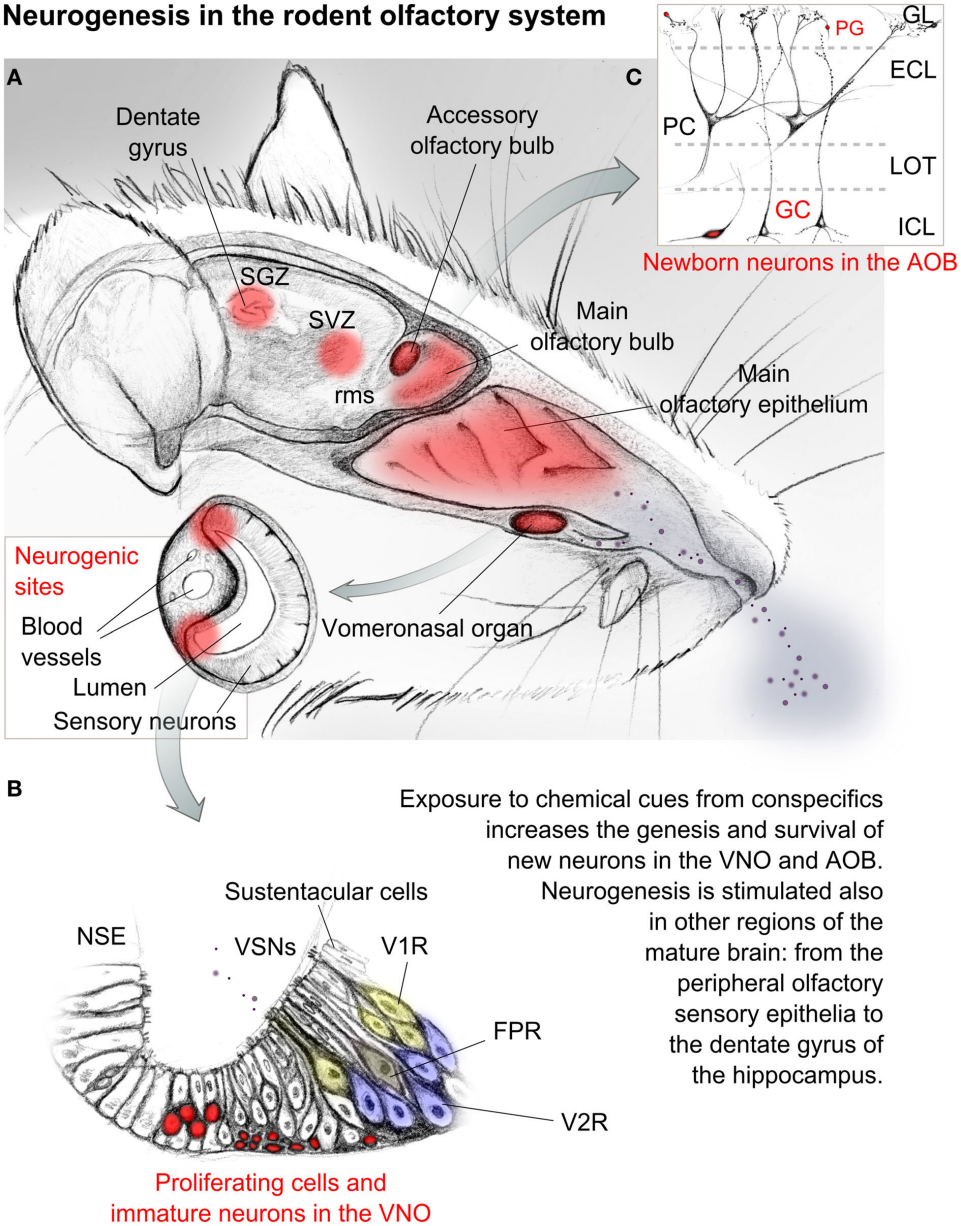
**Sketched representation of the mouse olfactory system**. Central and peripheral neurogenic regions are evidenced in red. **(A)** Social odors and chemical cues are detected through the main olfactory epithelium and the vomeronasal organ, which is enclosed in a bony capsule opened rostrally toward the nasal cavity. A highly vascularized cavernous tissue flanking the organ allows tissue contraction and therefore the access of mucuous fluids transporting chemical cues toward the sensory epithelium. **(B)** Enlarged view of the vomeronasal sensory epithelium and its cell types. Proliferating cells are localized at the lateral and basal margins of the matured sensory epithelium. Sensory neurons here located send axonal projections to the accessory olfactory bulb. **(C)** Simplified anatomy of the accessory olfactory bulb cellular layers. Cells evidenced in red in **(B,C)** represent immature or regenerating neurons. Abbreviations: SGZ, subgranular zone; SVZ, subventricular zone; rms, rostral migratory stream; GL, glomerular layer; PG, periglomerular cell; ECL, external cellular layer; ICL, internal cellular layer; LOT, lateral olfactory tract; PC, principal cell; GC, granule cell; V1R, vomeronasal receptor neuron type1; V2R, vomeronasal receptor neuron type2; FPR, formyl peptide receptor neuron; VSNs, vomeronasal sensory neurons; NSE, non-sensory epithelium.

Neurogenesis in the OB has been studied predominantly in the main olfactory pathway. The neurons constantly replaced in the main olfactory bulb (MOB) are GABAergic local interneurons (periglomerular, PGs, and granule cells, GCs) mainly derived from the Dlx2 subpallial domain in the SVZ (Puelles et al., 2000; Alvarez-Buylla and Garcia-Verdugo, 2002; Lledo and Gheusi, 2006; Whitman and Greer, 2009). These cells play a key role in regulating MOB input and output activity (Spors et al., 2012), and they have been proved to actively contribute to olfactory processing (Mandairon et al., 2011; Alonso et al., 2012) given their activity dependent survival and functional recruitment (Magavi et al., 2005; Mouret et al., 2008; Sultan et al., 2011a,b). In most of these reports the role of newborn neurons in the context of olfactory discrimination, short and long-term olfactory memory has been analyzed using synthetic odor compounds or artificial behavioral tasks. These paradigms are well suited to answer specific questions about the logic of sensory transduction (e.g., tuning, discrimination, detection threshold). However, framing the same analysis within the contexts of reproduction and sociality may be more informative to clarify whether neurogenesis itself is necessary or not to the mature brain. Indeed reproduction and sexual selection constitute a powerful evolutionary force and therefore the primary drive for any functional adaptation of a brain circuit. So far only few recent studies correlated MOB neurogenesis, with the regulation of social behavior in mice (see for example Larsen et al., 2008; Kageyama et al., 2012; Monteiro et al., 2013). The functional studies on the role of neurogenesis in the VNS are considerably fewer despite the major contribution of the VNS in rodent sociality (Tirindelli et al., 2009; Mucignat-Caretta, 2010; Chamero et al., 2012; Ibarra-Soria et al., 2013). Moreover the presence of neurogenesis in the AOB has been largely ignored, if not denied (Mak et al., 2007). However, neurogenesis occurs postnatally both at the VNS periphery, in the VNO, and more centrally, in the AOB (VNO: Barber and Raisman, 1978; Graziadei and Monti-Graziadei, 1979; Jia and Halpern, 1998; Giacobini et al., 2000; Martinez-Marcos et al., 2005; Weiler, 2005; Brann and Firestein, 2010; Enomoto et al., 2011; AOB: Bonfanti et al., 1997; Martínez-Marcos et al., 2001; Peretto et al., 2001; Huang and Bittman, 2002; Oboti et al., 2009, 2011; Figure 1). Interestingly cell survival in the AOB is higher after sensory stimulation around weaning and puberty onset (ca. 4 weeks in mice) when, after gonadal and endocrine maturation, social and reproductive behaviors become more clearly manifest (Oboti et al., 2011). Concurrently, despite the VNO seems to be already functional at birth (Coppola and O'Connell, 1989), the process of wiring and synaptogenesis of the VNO-AOB circuit has been shown to extend postnatally and to reach maturity only around the third postnatal week (Horowitz et al., 1999).

This seems to suggest the occurrence of a post-pubertal functional tuning of the VNS circuitry through neurogenesis, plasticity and constant rewiring, which goes beyond an early postnatal maturation of the system, similarly to the main olfactory system (Figure 1; Bonfanti and Peretto, 2011; Lepousez et al., 2013).

**Figure 2:**
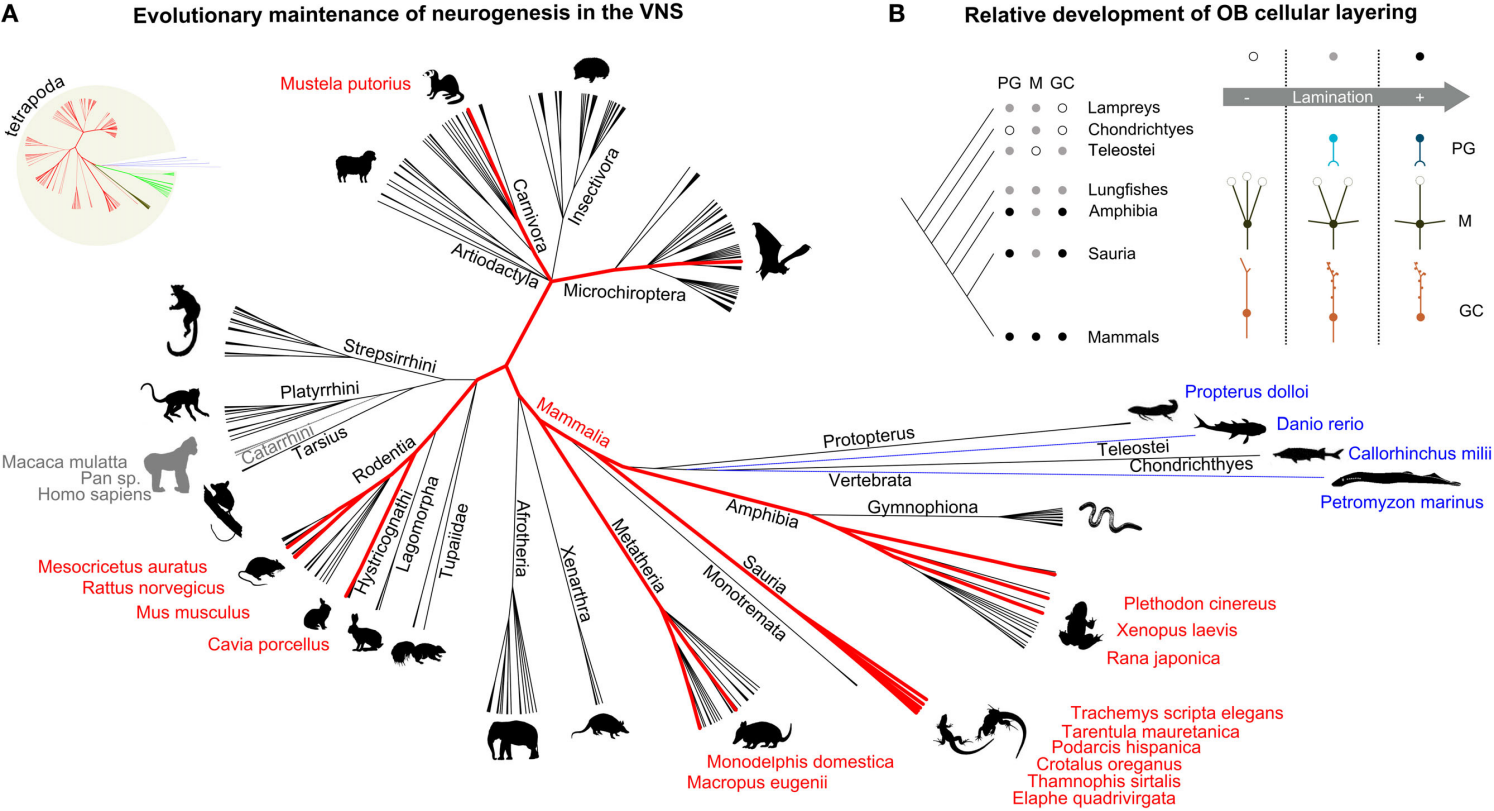
**Neuronal plasticity in the vomeronasal system is a conserved trait across several vertebrate species**. The increased organizational complexity of the olfactory systems is plausibly related to their protracted development through postnatal neurogenesis. **(A)** Cladogram representing the main vertebrate taxa in which the presence of a vomeronasal system has been reported (black lines). Taxa indicated in blue possess the cellular and molecular elements of the VNS, without a defined structural organization. Old world monkeys are indicated in gray, as reference, although they generally do not possess a functional VNS. The species (and related taxa) in which neurogenesis in the VNS has been reported are highlighted in red. **(B)** Features of the vertebrate olfactory systems include: more defined cellular layering (white: low, gray: moderate, black: high lamination), presence of periglomerular cells, PG (white: absent, gray: ambiguous, black: present), development of mitral cell secondary dendrites, M (white: absent, only multiglomerular primary dendrites, gray: presence of both multiglomerular primary dendrites and secondary dendrites, black: monoglomerular primary dendrites and secondary dendrites), loss of granule cell axon, GC (white: smooth dendrites, axon, gray: spiny dendrites, axon, black: spiny dendrites, no axon). Sources: (Meisami and Bhatnagar, 1998; Eisthen, 2000; Halpern and Martinez-Marcos, 2003; Grus and Zhang, 2008; Eisthen and Polese, 2009; Mucignat-Caretta, 2010); NCBI.

The reason why the VNS has been neglected by more recent studies on olfactory neurogenesis is possibly due to two main reasons. Firstly, the VNS is absent in humans, therefore limiting the interest in extending the study of olfactory neurogenesis to this system in rodents. Secondly, this sensory system has been traditionally associated to *pheromone* detection, innate signal processing and stereotyped endocrine responses, for which plasticity, neurogenesis, and rewiring are apparently not necessary. Nonetheless, regardless of any homologies in the mammalian olfactory systems, we undoubtedly share with rodents and other species the functions that this sensory pathway specifically regulates, when present (Figure 2). Therefore, one of the main reasons why neurogenesis in the rodent VNS deserves more attention is related to understanding the neural bases of mammalian neuroendocrine and behavioral development and how they are affected by environmental cues. Ultimately, the rodent VNS is a suitable and simple model to tackle wider issues related to other mammals in general, humans included.

The aim of the following paragraphs is to evaluate different aspects of VNS neurogenesis ranging from the phenotypes of newly generated neurons to their functional impact on specific circuits. The comparative description accompanying each of these points aims to open new questions for a wide range of approaches. These entailing the developmental, circuit, and system biology of olfaction. Aside, emerges the interesting—yet unanswered—question of how this olfactory subsystem acquires its function in the precise way it does, while its circuits are constantly changing. Rather than supporting the hardwired nature of this process, the evidences here reported suggest a necessary role for neurogenesis, neuronal plasticity, and environmental adaptation for its accomplishment.

## Neurogenesis in the vomeronasal organ

Olfactory sensory neurons (OSNs) are directly exposed to the environment to detect chemical stimuli through membrane bound receptors on their cilia (MOS) or microvilli (VNS). In the olfactory epithelia, ciliated and microvillus neurons, supporting cells and ensheating glial cells are constantly renewed during pre- and postnatal development (Murdoch and Roskams, 2008) by neural stem cells deriving from both neural crest and olfactory placode precursor lineages (Katoh et al., 2011; Heron et al., 2013; Suzuki et al., 2013). Due to this peripheral localization, OSN renewal has been generally associated to tissue growth (during development), homeostasis and repair (during adulthood) as gene expression patterns are maintained very similar (Heron et al., 2013). In the VNO, as in the MOE, the proliferation of different subsets of neuronal progenitors gives rise to OMP-positive mature receptor neurons (Murdoch and Roskams, 2008; Enomoto et al., 2011) but begins slightly later. In the rat olfactory epithelium OMP starts to be expressed at E14, while in the VN epithelium it occurs only at P2 (Kulkarni-Narla et al., 1997). In the mouse OMP is expressed in vomeronasal sensory neurons (VSNs) a few days earlier, during the last week of gestation (Tarozzo et al., 1998). These data indicate that VSNs are not fully developed at birth as most of their maturation begins and occurs postnatally.

Mature VSNs can be divided in three main families, depending on the receptors expressed and the ligands they have been reported to detect: V1Rs, activated preferentially by low molecular weight (LMW) hormone metabolites and other small molecules possibly contained in urine and bodily secretions; V2Rs, activated mainly by high molecular weight (HMW) peptidic compounds such as lipocalins, or smaller MHC peptides; formyl peptide receptors (FPRs), involved in the immune cell response to infections (Chamero et al., 2012; Figure 1B). V1R expressing neurons populate the apical portion of the sensory epithelium, V2Rs are located more basally while FPRs are more heterogeneously distributed (Rivière et al., 2009).

The earlier steps of VSNs differentiation are regulated by the proneural bHLH genes Mash1 and Neurogenin1, the former maintaining stem-cell progenitors, the second determining their multipotency (Cau et al., 2002), which is further regulated by the gene Ctip2 (Enomoto et al., 2011). Accordingly, loss of Ctip2 shifts the V1R/V2R differentiation ratio toward the V1R phenotype, suggesting a pivotal role in VSNs maturation and the possibility that the V2R-lineage entails both V1R and V2R committed neurons (Enomoto et al., 2011). The molecular mechanisms specifying the FPR lineage are not known.

VSNs proliferation is not homogeneous across the sensory epithelium but seems to be increasingly more localized at its margins, as development proceeds (Barber and Raisman, 1978; Giacobini et al., 2000; Martinez-Marcos et al., 2005; de la Rosa-Prieto et al., 2011). Although immature VSNs show limited migratory capabilities during adulthood (Martinez-Marcos et al., 2005; de la Rosa-Prieto et al., 2011), their proliferation increases until 2 months of age, in mice (Weiler, 2005; Brann and Firestein, 2010). Newborn VSNs are produced in clusters giving rise to patterned waves of migrating neurons (as observable by DCX immunohistochemistry). It would be interesting to clarify whether neurons expressing receptors of the same family are simultaneously generated at a given time. However, BrdU experiments suggested that both V1Rs and V2Rs are produced at the same pace in physiological conditions (de la Rosa-Prieto et al., 2010). Interestingly, postnatal development and growth seems to be present also in the non-sensory epithelium (NSE, Figure 1B) of the VNO (Garrosa et al., 1998; Elgayar et al., 2013).

Despite the vast number of MOE receptor genes (ca 1500 in the mouse) each OSN expresses only one of them. In the VNO this rule is not followed since each VSN may express more than one receptor gene (Martini et al., 2001; Silvotti et al., 2007; Ishii and Mombaerts, 2011). Interestingly, the phenotypic identity of newborn VSNs can be affected by histone modifications following application of urine ligands *in-vitro* (Xia et al., 2010). While application of HDAC inhibitors to cultured VSNs decreases the expression of immature neuronal marker Nestin, and increases the expression of markers of differentiation such as Map2, Neuro D1-D2, and V2R genes (Xia et al., 2010). It remains to be clarified whether these effects, represent a generalized adaptive response of the VNO epithelium to sensory stimulation or if it constitutes a mechanism to selectively specific subsets of newborn VSNs, as shown in the MOE (Watt et al., 2004; Dias and Ressler, 2013). Both simple parsimony and recent evidences suggest that this latter might be the case (Broad and Keverne, 2012). However, other factors such as odor exposure (Xia et al., 2006), hormonal changes (Kaba et al., 1988; Paternostro and Meisami, 1996) and sensory activity (Hovis et al., 2012) contribute to postnatal VNO neurogenesis and VNO-AOB rewiring indicating the persistence of constant adjustments in this circuit. Altogether these results strongly suggest that VNO neurogenesis do not serves mere tissue homeostasis and repair, but actively contributes to the functional tuning of the organ during postnatal development. A possibility still largely unexplored.

## Neurogenesis in the accessory olfactory bulb, a comparative note

Afferent axons from VSNs reach the brain at the level of the dorsal part of the OB. Here they form glomerular-like structures with the apical dendrites of a separate group of projection neurons which constitute the accessory olfactory bulb (AOB; Figures 1A,C). As for the MOB, their activity is regulated by inhibitory glomerular and granule cells (Figure 1C). Neurogenesis in the AOB involves mainly these two cell types (Oboti et al., 2009). Despite earlier doubts on its presence, several lines of evidence support the idea that adult neurogenesis represents a constitutive feature of the AOB. It can be found in adult mice of both genders (Oboti et al., 2009; Nunez-Parra et al., 2011), in adult rats (Peretto et al., 2001) and rabbits (personal observation). In addition, AOB neurogenesis has been reported not only in mammals (Altman and Das, 1966; Altman, 1969; Hinds, 1968a,b; Kaplan and Hinds, 1977; Bayer, 1983; Kaplan, 1985; Kishi, 1987), but also in other vertebrate species such as amphibians (Fritz et al., 1996), and reptiles (Garcia-Verdugo et al., 1989; Pérez-Cañellas and García-Verdugo, 1996; Pérez-Cañellas et al., 1997) indicating that it represents a conserved trait across different taxa (Figure 2A), rather than a parallel convergence. Neuronal plasticity in the olfactory system occurs independently of the presence of a discernible VNS, as in primates, fishes, cetaceans, and birds for example (García-Verdugo et al., 2002; Mucignat-Caretta, 2010); Figure 2A). In addition, taxa in which some of the typical cellular and molecular elements of the VNS are present, although with different levels of organization (as anurans, lungfishes, sea lampreys, teleosts, and cartilaginous fishes; Eisthen and Wyatt, 2006; Figure 2A), retain neuronal plasticity and neurogenesis in the primary olfactory structures during post-hatching and more mature stages. This indicates that a plastic VNS may not be an apomorphic (underived) trait of terrestrial vertebrates (Figure 2A; Table 1). In bat species, even though the VNS is not always developed, the presence of immature neurons typically expressing doublecortin (DCX) has been noted in the AOB (Amrein I., personal communication).

**Table 1:** **List of representative studies explicitly focused on VNO and AOB neurogenesis**.

**Species**	**VNO**	**AOB**
Mouse (*Mus musculus*)	Barber and Raisman, 1978	Hinds, 1968a,b
	Monti-Graziadei, 1992	Bonfanti et al., 1997
	Cappello et al., 1999	Oboti et al., 2009–2011
	Giacobini et al., 2000	Veyrac and Bakker, 2011
	Weiler, 2005	Sakamoto et al., 2011
	Martinez-Marcos et al., 2005	Nunez-Parra et al., 2011
	Murdoch and Roskams, 2008	
	Brann and Firestein, 2010	
	Enomoto et al., 2011	
Rat (*Rattus norvegicus*)	Monti-Graziadei, 1992	Altman and Das, 1966
	Weiler et al., 1999a–2005	Altman, 1969
	Inamura et al., 2000	Kaplan and Hinds, 1977
	Martínez-Marcos et al., 2000	Kishi, 1987
	Matsuoka et al., 2002	Bayer, 1983
		Peretto et al., 2001
		Corona et al., 2011
		Portillo et al., 2012
Rabbit (*Oryctolagus cuniculus*)	Othman, 2011	Personal observation
Guinea pig (*Cavia porcellus*)		Personal observation
Hamster (*Mesocricetus auratus*)	Ichikawa et al., 1998	Huang and Bittman, 2002
	Taniguchi and Taniguchi, 2008	
Opossum (*Monodelphis domestica*)	Jia and Halpern, 1998	Shapiro et al., 1997
	Shapiro et al., 1997	Martínez-Marcos et al., 2001
Wallaby (*Macropus eugenii*)	Ashwell et al., 2008	Ashwell et al., 2008
Ferret (*Mustela putorius furo*)	Weiler et al., 1999b	
*Bat (various* spp.)		Amrein et al., 2007 (OB)
Garter snake (*Tamnophis sirtalis*)	Wang and Halpern, 1982	
Striped snake (*Elaphe quadrivirgata*)	Kondoh et al., 2012	
Wall lizard (Podarcis hispanica)		Garcia-Verdugo et al., 1989
		Sampedro et al., 2008
		Font et al., 2012
Red-eared slider (*Trachemys scripta elegans*)		Pérez-Cañellas et al., 1997
Gecko (*Tarentola mauritanica*)		Pérez-Cañellas and García-Verdugo, 1996
Clawed frog (*Xenopus laevis*)		
No	Hansen et al., 1998	Fritz et al., 1996
	Higgs and Burd, 2001	
Yes	Endo et al., 2011	
Salamander (*Plethodon cinereus*)	Dawley et al., 2000	
	Dawley and Crowder, 1995	
Japanese brown frog (*Rana japonica*)	Taniguchi et al., 1996	
*Zebra fish (Danio rerio)*		Byrd and Brunjes, 2001*(OB)*
		Adolf et al., 2006 (OB)

*Species in which neurogenesis in the VNS components of the olfactory system could be present are indicated italics*.

In mice, rats, rabbits and guinea pigs SVZ neuronal progenitors give rise to neuroblasts integrating in the AOB mainly as granule cells localized in the inner granule cell layer, below the lateral olfactory tract. The presence of newborn neurons in periglomerular layer is very limited, possibly reflecting a much slower turn-over rate of PGs (Martínez-Marcos et al., 2001; Peretto et al., 2001; Oboti et al., 2009; Nunez-Parra et al., 2011). A limited number of cells can be found in the plexiform layer between the GC and PG layers, where principal cells (PC) are located (Oboti et al., 2009; PCs are homologous to MOB mitral cells), possibly representing other cell types such as external granule cells and dwarf cells (Larriva-Sahd, 2008). The increasing importance of local interneurons for OB signal elaboration, reflected by the increased complexity in OB structural lamination (Eisthen and Polese, 2009; Figure 2B), implies a possible correlation with the maintenance of their turnover. Overall, OB neurogenesis may represent a necessary and conserved feature of the olfactory pathways, reminiscent of the higher neuronal plasticity showed by the paleocortex, to which it belongs (see Table 1 for a list of representative studies explicitly focused on VNO or AOB postnatal development and neurogenesis). In the following paragraphs, attention will be given to the anatomy of the AOB, the phenotypes of AOB newborn cells and the possible role in its circuitry considering the present knowledge about its role in the VNS.

## Neurogenesis in the two accessory olfactory bulb subregions

Both the glomerular and principal cell (PC) layer of the AOB look clearly partitioned by the segregated V1R/Gai2 and V2R/Gao afferent fibers. Axonal projections from these two neuronal populations establish synaptic contact with either the anterior (aAOB) or posterior (pAOB) AOB, respectively. This separation is visible in the PC layer neuropil (*linea alba*, Larriva-Sahd, 2008). In rodents the V1R and V2R pathways have been shown to selectively respond to low molecular weight organic molecules (Leinders-Zufall et al., 2000; Sugai et al., 2006) and high molecular weight compounds of peptidic nature, respectively (Leinders-Zufall et al., 2004, 2009; Kimoto et al., 2005). Accordingly to this functional dichotomy, differences in c-Fos expression patterns in the two AOB regions have been observed after exposure to gender related odors in male and female mice (Kumar et al., 1999; Halem et al., 2001). Interestingly, in mice and rats (Peretto et al., 2001; Oboti et al., 2009, but not in opossums Martínez-Marcos et al., 2001), newborn cells reaching the AOB in physiological conditions (no odor exposures) seem to be unequally distributed along the rostro-caudal axis. This may reflect a differential rate of development of the two AOB sub-regions or alternatively be related to the V1R/V2R functions being subjected to differential adaptive pressures in a given eco-ethological niche (Suárez et al., 2011a,b). Accordingly, recently a dual embryonic origin of the AOB has been proposed (Huilgol et al., 2013). In this study, Huilgol and coauthors showed that PCs in the pAOB derive from the thalamic eminences at the diencephalic/telencephalic boundary (DTB) from Lhx5 expressing neurons, as part of the amygdala, BST and Cajal-Retzius neurons (Huilgol et al., 2013), while the aAOB PCs share common origin with MOB mitral cells, as indicated by Tbx21 expression in the OB primordium (Huilgol et al., 2013). The DTB is evolutionary conserved in amphibians and mammals indicating that the pAOB may be a residue of the earliest sensory systems originating from the thalamic eminences and controlling olfactory responses in amphibians (Krug et al., 1993; Huilgol et al., 2013). However, the apparent morphology of AOB granule cell layer does not reveal a similar dichotomy, as AOB granule cells connectivity may be more ambiguous (Larriva-Sahd, 2008). Despite the different origin of pAOB projection neurons, immature GABAergic interneurons of both the aAOB and pAOB may include cells derived from the same Dlx2/5/6, Emx1, and Meis2 lineages in the SVZ (Kohwi et al., 2007; Agoston et al., 2013). Overall this suggests the existence of different regulatory mechanisms locally specifying the phenotype of cells belonging to the same neuronal lineage but integrating in different circuits (aAOB vs. pAOB, but also AOB vs. MOB). Further understanding of the underlying mechanisms would extend our knowledge of the morphological and functional adaptations newborn neurons may be capable of. In addition, given the functional segregation of the VNS circuits (V1R-aAOB, V2R-pAOB), different levels of neuronal plasticity and neurogenesis may reflect a different degree of adaptability to the diversity of chemical stimuli each subsystem elaborates. However, despite the differences these features may have a similar functional relevance for their proper function.

## Phenotypes of newborn neurons in the accessory olfactory bulb

New neurons migrating from the SVZ through the rostral migratory stream, reach all AOB layers: glomerular (Gl), external (ECL), and internal (ICL) cellular layers (Larriva-Sahd, 2008). At present no detailed analysis has been made to identify these cell types. Moreover, the presence in the MOB of newly generated tbr2-derived glutamatergic cells (Brill et al., 2009) and GABAergic-serotonergic (Inta et al., 2008) interneurons has been recently proven, while in the AOB it remains to be verified.

In the MOB, the cell types forming the glomerulus (juxtaglomerular cells) are classified in periglomerular (PG), short axon (SA) and external tufted (ET) cells, based on their neurochemistry, morphology, and connectivity. Juxtaglomerular cells can be divided in two main GABAergic chemotypes based on the expression of different isoforms of the GABA synthesis enzyme—GAD-65, GAD-67—together with other markers such as dopamine (DA), or its synthesis enzyme tyrosine hydroxylase (TH), calbindin, calretinin, and others (Shipley et al., 2004). Virtually all dopaminergic neurons express GAD-67, while little if no overlap is present between the GAD-65 and the TH sub-populations (Kiyokage et al., 2010). As typical SA cells, TH-GAD-67 neurons innervate multiple glomeruli while GAD-65 neurons are mostly monoglomerular with only few secondary processes directed to other glomerular formations (Aungst et al., 2003; Kiyokage et al., 2010).

In the AOB glomerular layer scarce if not absent GAD-67 staining has been reported together with almost complete lack of TH expression (Mugnaini et al., 1984; Oboti et al., 2009). This possibly indicates a predominance in the AOB of the monoglomerular GAD-65 chemotype. However, newly generated cells with morphological features of both PG uniglomerular cells and SA multiglomerular cells have been identified in the AOB (Oboti et al., 2009) suggesting that the low levels of GAD-67 expression do not necessarily imply the absence of SA-like cells (Mugnaini et al., 1984; Larriva-Sahd, 2008).

In the MOB, dopaminergic PG cells are responsible of thresholding mitral cell firing in response to olfactory inputs (Pírez and Wachowiak, 2008). The lack of dopaminergic signaling in the AOB may imply a minor need for gain control of vomeronasal inputs on PCs being their firing threshold possibly determined by input coincidence from heterotypical glomerular afferents (Meeks et al., 2010).

Other inhibitory cells located more deeply in the AOB are external and internal granule cells, located above and below the lateral olfactory tract (LOT), respectively. Evidence showed the vast majority of newborn cells reaching the AOB is represented by internal granule cells (Oboti et al., 2009; Nunez-Parra et al., 2011). In the ICL main accessory cells are also present (MACs, Larriva-Sahd, 2008) and are distinguishable from granule cells by larger soma and nuclear size and by their sporadic presence in the LOT. Although newborn cells can be often found in the LOT, their nuclear size was always comparable to normal granule cells (external granule cells, in this case), thus limiting the likelihood for MACs to be regenerated during adulthood.

Recently, in the rat MOB the presence of newborn neurons in the external plexiform layer (EPL) has been proved (Yang, 2008). These neurons have been reported to be PV/CR expressing Van Gehuchten cells, multipolar cells and superficial SA cells (Yang, 2008). Since DCX- and BrdU-positive cells can be found in homologous locations in the AOB (ECL), the presence here of these cell types is possible but yet to be investigated.

It is not known whether SVZ-derived interneurons migrating to the AOB belong to the same lineage of those in the MOB. It is possible that genetically distinct populations of interneurons are heterogeneously distributed in the two OB sub-regions. Recently, viral fate-mapping experiments revealed the mosaic nature of the SVZ proliferative domains giving rise to different and heterogeneous pools of GABAergic interneurons (Merkle et al., 2007). However, upon inspection of the material used in this study, no apparent regionalization of either aAOB- or pAOB-committed progenitors was found (viral infected GFP+ cells were found in the AOB of mice injected at all SVZ levels, personal observation). Interestingly, although most of newborn AOB neurons labeled with BrdU coexpress NeuN at 4 weeks of age (80%) as in the MOB, the level of coexpression with other interneuronal markers is much lower (BrdU/GABA, BrDU/GAD-67, and BrdU/calretinin reach only about the 30%; Oboti et al., 2009) (MOB: BrdU/GAD67 is about 80% in the GrL and 30% in the GL; Parrish-Aungst et al., 2007). These results indicate that the phenotype of AOB newborn neurons is similar to the MOB but conserve some peculiarities specified either locally or in the SVZ. The different relative abundance of morpho- and chemo-types in this structure, renders the AOB an interesting circuit to study the differential role of a given cell type in different compartments of the bulbar circuitry. For example by studying PC (AOB mitral cell homolog) electrical responses to peripheral nerve stimulations it would be possible to clarify to which extent SA cells in the AOB may be dispensable—in case of their limited presence in this structure—for a certain olfactory coding task, or—by comparison—which specific function do they serve when present in other bulbar circuits.

## Sensory activity-dependent survival and function of newborn cells

As shown by olfactory enrichment or deprivation studies, the maturation and survival of newborn neurons in the MOB depends on sensory inputs (Cummings et al., 1997; Rochefort et al., 2002; Mandairon et al., 2006). Newborn neurons reach the MOB in massive waves but are gradually selected during integration into local circuits (Petreanu and Alvarez-Buylla, 2002) activated by sensory inputs (Magavi et al., 2005; Mouret et al., 2008; Sultan et al., 2011a,b). Importantly, loss or ablation of newborn neurons in the MOB can impair olfactory function (Breton-Provencher et al., 2009; Mandairon et al., 2011) since younger cells are preferentially involved in these circuits (Nissant et al., 2009; Alonso et al., 2012).

Although renewing at a slower rate (Oboti et al., 2009), newborn cells in the AOB are likely to similarly contribute to VNS function. As in the MOB, sensory activity increases the survival of newborn neurons in the AOB (Oboti et al., 2009, 2011; Nunez-Parra et al., 2011). This effect is mediated by chemical cues present in urine or bodily secretions (Nunez-Parra et al., 2011; Oboti et al., 2011), it is abolished after VNO genetic functional ablation in trpc2-ko mice (Oboti et al., 2011), it is persisting until 7 months of age (Nunez-Parra et al., 2011), and gives rise to neurons responding preferentially to experienced odor stimuli (Oboti et al., 2011).

The presence of gender related differences in AOB neurogenesis is controversial (no differences in CD1 mice Oboti et al., 2009, 2011; differences in B6 mice, Nunez-Parra et al., 2011). However, the effect of odor experience on AOB neurogenesis seems to be particularly evident in post-pubertal female mice after male odor stimulation (Oboti et al., 2009, 2011; Nunez-Parra et al., 2011). This seems particularly evident in the aAOB upon chronic exposure to low molecular weight (LMW) chemical cues present in male urine, which are mainly detected by the V1R neurons (Oboti et al., 2011). Larger protein compounds sensed through V2Rs are instead ineffective on neuronal survival in neither of the two AOB regions (Oboti et al., 2011). However, a lack of increase in surviving cells after sensory enrichment does not necessarily imply the absence of a sensory dependent functional recruitment of newborn elements, but only that the net amount of surviving cells remains stable. Considering that social odors are important primers on mice development and reproductive behavior, these findings suggest a possible role of AOB postnatal/adult neurogenesis in sensory processing in both genders. Accordingly, eliminating newborn cells in the whole bulb, AOB included, Sakamoto and colleagues showed for the first time an impairment in olfactory functions involving the VNS such as predator-odor avoidance, aggression and mounting in males (Sakamoto et al., 2011). A finding that has been extended to VNO-dependent mate recognition in females (Oboti et al., 2011). The effect of sensory inputs on AOB neurogenesis overall indicates that newborn neurons play an active and possibly relevant role on the vomeronasal circuitry during postnatal and adult life.

## Impact of newborn neurons on AOB circuits

A few comparative considerations with the MOB elementary functional unit—the olfactory column—can be insightful in defining the impact of newborn neurons on AOB network activity. The MOB olfactory column is considered equivalent to the cortical columns and barrels in the visual and somatosensory cortices (Shepherd, 2010). It comprises all OSNs projecting to a single glomerulus, all the mitral and tufted cells extending their dendrites to it and all the granule cells connected to these projection neurons. Granule cells can regulate mitral/tufted cell output providing self inhibition through dendrodendritic synapses on mitral cell lateral dendrites within the same column. In addition, they may exert lateral inhibition on adjacent columns by shunting the propagation of action potentials on distal lateral dendrites of extra-columnar mitral cells (Xiong and Chen, 2002). This implies a dual role of granule cells on mitral/tufted cell firing: through self-inhibition within the same column, granule cells may act synchronizing the firing rate of projection neurons belonging to different units while responding to the same sensory input (Dhawale et al., 2010); through lateral inhibition on extracolumnar mitral cells, granule cells may provide contrast enhancement between two different functional units (as other amacrine—axonless—cells in the retina for instance; Migliore and Shepherd, 2008). Both effects have been hypothesized to be relevant for olfactory discrimination (Migliore and Shepherd, 2008; Dhawale et al., 2010; see Lepousez et al., 2013 for a detailed review on this hypothesis). Given the apparent lack of columnar organization in the piriform cortex, this topological motif in the bulbar circuitry probably reflects its cortical like function and represents the modular unit encoding the diversity of olfactory inputs (Haberly, 2001; Migliore et al., 2007). Importantly, the constant re-adjustment of the synaptic inputs caused by renewal of both local interneurons and olfactory fibers has been associated with an optimization of this function (Alonso et al., 2006; Jones et al., 2008; Adam and Mizrahi, 2011).

The AOB appears structurally similar to the MOB, although it retains some peculiar features in both hodology and cell types. However, the occurrence of similar plastic events in both structures motivates the same reasoning done for the MOB. Olfactory glomeruli in the AOB are on average smaller than those in the MOB and appear to be clustered in pseudostratified formations. Contrarily to MOB glomeruli, they receive multiple inputs from different types of VSNs (Takami and Graziadei, 1991; Belluscio et al., 1999; Del Punta et al., 2002), with V1Rs projecting only to the aAOB and V2Rs to the pAOB. In addition, neurons expressing the same receptor/s in the VNO, may project to up to 20–30 different glomeruli (Belluscio et al., 1999), while same-receptor OSNs in the MOE project mainly to two symmetrical glomeruli in the MOB. This conserved pattern seems to underlie a higher degree of input convergence on MOB projection neurons and therefore functional specialization of each olfactory column in the MOB (Hildebrand and Shepherd, 1997; Su et al., 2009; Touhara and Vosshall, 2009) as mitral cells project to a single glomerulus, therefore receiving afferents from OSNs expressing the same receptor. Conversely, AOB PCs reach multiple glomeruli receiving inputs from different VSNs (a feature shared with the OB of fishes: Ngai et al., 1993; Speca et al., 1999). However, AOB projection neurons maintain V1R/V2R segregated apical dendritic arborizations depending on their location in the aAOB and pAOB (Jia and Halpern, 1997). Nonetheless, a cross talk may exist between aAOB and pAOB principal cells via thinner lateral dendrites crossing the midline (Larriva-Sahd, 2008). As a result of their heterotypic connectivity, AOB PCs integrate inputs from different receptor types in the VNO and therefore different ligands. Slice recordings on *ex-vivo* VNO-AOB intact preparation showed that this is indeed the case (Meeks et al., 2010). Juxtaglomerular complexes in the AOB resemble the functional triads described in the MOB: PG and SA cells have inter- and intra-glomerular projections, external tufted cells contact a single glomerulus.

The limited extent by which AOB PGs are regenerated by SVZ-derived progenitors, together with the above mentioned lack of TH, could be explained by the lack of TH/GAD-67 cells in the AOB. Alternatively, since TH expression levels in PGs are traditionally used as a proxy for olfactory input (Nadi et al., 1981; Baker et al., 1983; Cho et al., 1996) and VNO activity is subordinated to initial odor detection by the MOE (Xu et al., 2005; Slotnick et al., 2010), the lack of TH and GAD-67 in the AOB could be just a consequence of the irregular nature of vomeronasal inputs. The expression patterns of other activity markers (such as cytochrome-c-oxydase or β-secretase-1) in the AOB glomerular layer resemble those in the MOB during sensory deprivation and therefore could support this hypothesis (Yan et al., 2007; He et al., 2014).

Conversely, granule cells in the AOB are the most represented cell type among newly generated neurons (Oboti et al., 2009; Nunez-Parra et al., 2011). They are typically located in the deep ICL (below the LOT) but also in the deeper portion of the ECL and in the LOT, just below PC somata. They project to PC dendrites belonging to the homologous region (aAOB or pAOB), but considering that PC axon collaterals cross repeatedly the two sub-regions, they could receive synaptic inputs from both. In addition their apical dendrites seem to reach the glomerular layer (Larriva-Sahd, 2008), although it is not clear whether they interact synaptically with the juxtaglomerular complex. Interestingly, while EGC dendritic processes appose on PC somata or proximal dendrites, those from IGC seem to localize preferentially on distal and apical processes, between glomeruli and PC somata (Larriva-Sahd, 2008), a feature confirmed by EM studies (Moriya-Ito et al., 2013). This distinction may imply a bias for AOB IGCs toward PC self-inhibition, instead of intercolumnar lateral inhibition. Eventually, since the vast majority of newly generated cells in the adult AOB are IGC, it is appealing to imagine neurogenesis in the AOB as a mechanism to shunt directly input signals from the VNO. Given the variable turnover rate of IGCs during postnatal development, this feature alone would be sufficient to justify changes in the response to vomeronasal sensory cues over time. In addition, given that both the survival and activation of newborn neurons is actively driven by vomeronasal sensory inputs, this selective shunting may contribute to encode stimulus familiarity. In general, a change in IGC turnover rate, together with other physiological changes, may set the timing for certain stimuli to be more or less effective as social signals or endocrine modulators (e.g., effect of male urine odors on female estrous varying depending on kinship or shared fostering). Ultimately, this could represent a possible answer to the question posed by the title of this manuscript.

Overall these observations suggest that newly generated GABAergic interneurons differently contribute to mature AOB circuits, if compared to the MOB. The different nature of GABAergic modulation of AOB output signals is further supported by the firing of AOB PCs, which appears to be longer sustained, if compared to MOB mitral cells (Meeks et al., 2010; Shpak et al., 2012). In addition, given PC heterogeneous glomerular connectivity and the convergence of their centripetal projections to more central targets (Salazar and Brennan, 2001), the information conveyed by their output signals is also different, and probably more complex. As a direct consequence and in the whole system perspective, the impact newborn IGCs have on PC output activity may definitely be higher than that of granule interneurons on MOB mitral cells.

## Conclusions

Overall the considerations made in this manuscript are meant to underline that the VNS is not only constitutively plastic but also that this plasticity may constitute the basis for its peculiar function. Eventually the VNS circuitry cannot be considered hardwired but rather able to adjust its connectivity to environmental changes. If the function of the VNS described so far (see for critical views on this point Eisthen and Wyatt, 2006; Mucignat-Caretta et al., 2012) is the result of the interaction between plastic circuits and environmental stimuli, is definitely not known and certainly deserves further investigation. Plausibly neuronal plasticity and neurogenesis are indeed necessary to shape it and maintain it throughout postnatal life. Eventually different rates of neurogenesis can determine the extent by which VNS circuits adapt and tune to a given chemical environment, being it referred to social, reproductive, or aggressive/territorial behaviors. Even though the VNS is not simply the *pheromone-detector* in the nasal cavity of mammals or other vertebrates (Eisthen and Wyatt, 2006), it would be a challenge of future studies to test the impact of neuronal plasticity and neurogenesis on those functions, commonly associated to *pheromone* sensing. Indeed studying the molecular and genetic mechanisms underlying the neuroendocrine physiology of sociality may yield insights on the etiology of associated anomalies, even in those mammalian species, human included, in which this sensory pathway is not present. For this reason the rodent VNS may represent the unique opportunity to dissect this issue in an animal model in which these features strongly rely on its functional integrity and—plausibly—its capability of cell renewal through adult neurogenesis.

### Conflict of interest statement

Figure 1A was adapted from published material, courtesy of Catherine Delphia. Cladogram in Figure 2B was adapted from material copyrighted by Elsevier, courtesy of Heather Eisthen and Elsevier. The authors declare that the research was conducted in the absence of any commercial or financial relationships that could be construed as a potential conflict of interest.

## Abbreviations

aAOB: anterior accessory olfactory bulb
AOB: accessory olfactory bulb
CR: calretinin
DCX: doublecortin
DG: dentate gyrus
ECL: external cellular layer
EGC: external granule cell
ET: external tufted
GAD: glutamic acid decarboxylase
GC: granule cell
Gl: glomerular layer
HMW: high molecular weight
ICL: internal cellular layer
IGC: internal granule cell
LMW: low molecular weight
LOT: lateral olfactory tract
MACs: main accessory cell
MOB: main olfactory bulb
MOE: main olfactory epithelium
MOS: main olfactory system
NSE: non-sensory epithelium
OB: olfactory bulb
OSNs: olfactory sensory neurons
pAOB: posterior accessory olfactory bulb
PC: principal cell
PG: periglomerular cell
PV: parvalbumin
SA: short axon cell
SGZ: subgranular zone
SVZ: subventricualr zone
TH: tyrosine hydroxylase
VNO: vomeronasal organ
VNS: vomeronasal system
VSNs: vomeronasal sensory neurons.
